# Protective Factors against Depressive Symptoms in Female American Indian Cancer Survivors: The Role of Physical and Spiritual Well-being and Social Support

**DOI:** 10.31557/APJCP.2021.22.8.2515

**Published:** 2021-08

**Authors:** Soonhee Roh, Yeon-Shim Lee, Yi-Ping Hsieh, Scott D. Easton

**Affiliations:** 1 *Department of Social Work, University of South Dakota, State of South Dakota, USA. *; 2 *School of Social Work, San Francisco State University, State of California, USA. *; 3 *Department of Social Work, University of North Dakota, State of North Dakota, USA. *; 4 *School of Social Work, Boston College, State of Massachusetts, USA. *

**Keywords:** American Indian women, cancer survivors, depressive symptoms, spiritual well-being, social support

## Abstract

**Background:**

This exploratory study examined how perceived physical well-being, spiritual well-being and social support relate to depressive symptoms among American Indian (AI) female cancer survivors.

**Methods:**

Cross-sectional data were obtained from 73 AI female cancer survivors between 32 to 77 years of age in the Midwest of the United States. Standardized measures were used to assess for depression (Center for Epidemiologic Studies Depressive Symptoms Scale Short Form), spiritual well-being (Functional Assessment of Chronic Illness Therapy, Spiritual Well-being Scale), and social support (Medical Outcomes Study of Social Support Questionnaire). A single item measured physical well-being. After univariate and bivariate analyses, hierarchical multiple regression (HMR) was used for modeling.

**Results:**

Approximately 47% of the sample scored higher than 10 on the depressive symptoms scale. HMR results indicated that perceived physical well-being, spiritual well-being, and social support were negatively associated with depressive symptoms, accounting for 47% of the variance in the final model (adjust R^2^ = 0.47).

**Conclusions:**

A high percentage of the sample exceeded the cut point for probable depression, highlighting the importance of addressing mental health in the aftermath of cancer treatments for AI women. Results suggest that perceived physical well-being, spiritual well-being, and social support serve as protective factors and possible levers to reduce depression in this population. Interventions designed to bolster existing social support and spirituality could improve the mental health of AI women in the aftermath of cancer treatment. Community-based, culturally appropriate health education programs should be developed to enhance AI women’s physical health.

## Introduction

Cancer is the number one cause of death for American Indian (AI) women (American Indian Cancer Foundation, 2021). Depression is often associated with cancer, with reported prevalence rates of depression ranging from 20% to 50% among cancer patients in the general population (Bower, 2008; Ell et al., 2005; Mitchell et al., 2011). Depression rates vary among cancer patients by geography and health care settings. In one study (Walker et al., 2013), rates ranged from 5 to 16% among outpatients, 4 to 15 % among inpatients, 4 to 11% for those in mixed outpatient settings, and 7 to 49% for palliative care patients. Additionally, depressive symptoms are among the most salient factors that determine quality of life for cancer patients (Saracino et al., 2017). However, few studies have examined the prevalence or longitudinal impact of this comorbidity on cancer survivors’ health outcomes (Bower, 2008; Ell et al., 2005; Mitchell et al., 2011). 

Research has focused on protective factors to bolster health among cancer patients in the general population. Many researchers have found that spiritual and physical well-being play significant roles in managing health among cancer survivors (e.g., Koenig et al., 2012). Similarly, the importance of social support in maintaining physical, emotional, and social well-being has drawn the attention of researchers in health disciplines. In particular, a growing number of studies indicate associations of social support with cancer survivors’ physical and mental health (Ganz et al., 2003; Helgeson and Cohen, 1996).

As a culturally heterogeneous group within the U.S. population, AIs are comprised of nearly 5.6 million people who are affiliated with 574 federally-recognized tribes (U.S. Department of the Interior Indian Affairs, 2021). Yet understanding of protective factors against depressive symptoms among AI cancer survivors is virtually non-existent. To fill this void in the knowledge base, the present study examined how perceived physical well-being, spiritual well-being and social support promote psychological resilience among AI female cancer survivors in terms of depressive symptoms. Psychological resilience has been defined as one’s ability to cope positively with stress or to maintain a positive outlook on life, despite negative circumstances (Connor and Davidson, 2003).

Perceived physical health status has been identified as a key factor influencing depression prognosis (Ambresin et al., 2014). Perceived physical health status, widely considered as an indicator of one’s general health, is often measured through a subjective, self-rating of present health status (Idler and Benyamini., 1997). Numerous studies documented that depressive symptoms are strongly linked to poor self-reported physical health, high functional disability, and more chronic disease (Ambresin et al., 2014; Jang et al., 2021). In a longitudinal study, self-reported health was found to be a significant predictor for recurrence of major depressive symptoms up to five years later (Ambresin et al., 2014). Although much research has documented the relationships between perceived health status and long-term mental health outcomes in cancer patients and the general population, little empirical research has been conducted with AI. Thus, it is important to explore the associations of perceived physical health status with depressive symptoms among AI female cancer survivors.

Available research indicates that spiritual well-being and AI healing practices are profoundly important for holistic healing and meaning for AI cancer survivors (Roh et al., 2018). Although spiritual practices may vary across 574 tribes, certain concepts are commonly held by AIs regardless of tribal affiliation, such as holistic views of health with an emphasis on balance and harmony. According to Marbella et al., (1998), 38% of AI patients in primary care settings used AI traditional healers, while 86% would seek one in the future. Another study indicated that AI populations commonly use spiritual and traditional health practices, with 70% of an urban AI sample in primary care setting reporting frequent usage (Buchwald et al., 2000). In a more recent study, spiritual well-being was highly related to cancer treatment and care in this population (Roh et al., 2018). Nonetheless, more research on protective factors related to depressive symptoms among AI cancer patients, such as perceived spiritual health, is needed. 

A third possible protective factor for quality of life among cancer survivors is social support, which can diminish the effects of life stressors (e.g., illness) through by strengthening survivors’ interpretation of the experience (Lee et al., 2018). Many researchers reported that social support systems are significant protective factors for AIs experiencing stressful life events (Henson et al., 2017; Lee et al., 2018; Roh et al., 2015). Although social support has been found to be helpful to AIs coping with cancer (Lee et al., 2018), only two articles specifically focused on social support among AI women cancer survivors (Bauer et al., 2005; Lee et al., 2018). Lee et al., (2018) conducted a study with 43 AI women who had survived cancer and found that they received support from friends, fellow cancer survivors and support groups, church members, community groups, and healthcare providers. In another study, Bauer et al., (2005) compared the social network topology of 40 AI cancer patients with 41 non-AI controls, but did not find significant differences across the groups. Interestingly, expressive social support (e.g., emotional support, companionship, and advice) was reported as most important by all, often through companionship from friends and spiritual support (e.g., prayers) from church members. Interestingly, instrumental support (e.g., financial, favors, and household maintenance), usually from family members, was not ranked as important (Bauer et al., 2005). The results of these studies shed some light on the inter-relational and cultural meanings of receiving support by cancer patients. While the association of social support with depressive symptoms has been well-established in cancer patients in general, less is known about the relationship among AI women cancer survivors. Given the lack of studies on AI women cancer survivors, the current study aimed at identifying protective factors (e.g., physical health, spiritual well-being, and social support) promoting resilience against depressive symptoms within this population. 

## Materials and Methods

*Data Collection and Participants*

After receiving approval from four Institutional Review Boards (i.e., university, two hospitals, and funding agency), a cross-sectional survey was administered to participants between June 2014 and February 2015. Within the exploratory quantitative methodology, we formed a community advisory board (CAB) comprised of healthcare professionals who work in two metropolitan areas of South Dakota (i.e., Sioux Falls and Rapid City) and leaders in the AI community. The CAB participated in all phases of the study, including community needs and concerns around research, recruitment, dissemination, and project evaluation. A purposive sampling was used according to recommendations from our CAB. Participants were recruited from two non-profit hospitals: Avera Medical Group Gynecologic Oncology in Sioux Falls and John T. Vucurevich Cancer Care Institute, Rapid City Regional Hospital. 

Inclusion criteria for participation were: (1) a personal history of any type of cancer within the previous 10 years; (2) a self-identification as an AI woman; (3) completion of cancer treatment without signs or symptoms of recurrence at the time of survey; and (4) 18 years of age or older. Participant recruitment involved three steps. First, staff at the two partner hospitals developed a list of cancer survivors (n=100) and then mailed a study announcement. Next, the first author conducted initial screenings by phone of people who responded to the advertisement and/or on the list (n=76). Three individuals were excluded during screening (i.e., more than 10 years had elapsed since cancer diagnosis), leading to the final sample of 73. After determining eligibility, a survey was then scheduled at participants’ preferred location (e.g., residence, private conference room in community church, and first author’s office).

Before administering the survey, the research team explained the purpose of our study and related issues (e.g., eligibility criteria, risks/benefits, confidentiality, voluntary nature of participation, right to withdraw). Participants provided written, signed informed consent, completed a self-administered survey, and were each compensated US$20.

*Measures*

Dependent variable. The Center for Epidemiologic Studies Depressive symptoms Scale Short Form (CES-D-SF) (Radloff, 1977) was used to examine depressive symptoms. The instrument consists of eight negatively stated items and two positively stated items (reverse coded) that assess frequency of symptoms (e.g., loneliness, fearfulness, sleep problems) during the past week. Item responses were based on a 4-point Likert scale. Scores ranged from 0 to 24, with higher scores indicating more depressive symptoms. Although a clinical diagnosis of depression cannot be made, the suggested cutoff for probable depression is 10 or higher. Internal consistency was .80 in the current study.

Spiritual Well-Being. Spiritual well-being were assessed with the Functional Assessment of Chronic Illness Therapy, Spiritual Well-being Scale 12 (FACIT-Sp12) (Cella et al., 1993). Developed in 1990, FACIT-Sp12 is a 12-item brief measure of spirituality with three domains: peace, meaning, and faith. FACIT-Sp12 was designed for self-administration and uses a 5-point Likert scale (0 = not at all; 1 = a little bit; 2 = somewhat; 3 = quite a bit; and 4 = very much). Scores range from 20 to 48, with higher scores indicating higher levels of spiritual well-being. Internal consistency was .76 in the current study.

Social Support. The Medical Outcomes Study Social Support questionnaire (MOS-SS; Sherbourne and Stewart, 1991) was used to measure perceived social support. The 10-item questionnaire assesses availability of five dimensions of social support: positive social interactions, emotional support, informational support, tangible support, and affectionate support. Participant responses are based on a five-point Likert scale ranging from (1) seldom to (5) always. Scores on the social support measure can range from 10 to 50, with higher scores indicating greater support. Internal consistency was .95 in the present study.

Perceived Physical Well-being. Perceived physical well-being was measured using one item: “how would you rate your overall health at the present time?” Responses were based on a four-point Likert scale ranging from (1) poor to (4) excellent.

*Analytical Plan*

To examine the sample’s characteristics and the study variables, descriptive analysis were conducted. We also conducted correlational analysies using the Pearson coefficient of correlation to examine interrelationships between study variables. Hierarchical multiple regression (HMR) was performed to test associations of physical and spiritual well-being and social support with depressive symptoms. We first entered perceived physical well-being into the model, followed by spiritual well-being in the second model and social support in the third model. This allowed us to incrementally assess predictive power of each concept above and beyond demographic factors. 

## Results

*Descriptive Statistics*

The mean age of the AI female cancer survivors was 56.49 years with a standard deviation of 11.18 (range = 32-77). Most participants (68.5%) had some college/associate degree or higher; 28.8% held high school degrees, and 2.7% had middle school degrees. For household income, 22.2% of participants reported monthly family earnings of $3,000 or more, 32% reported $1,000 - $2,000 per month, and 26.4% earned less than $1,000 per month. At the time of the survey, about 38.4% of the participants were divorced, 30.1% were either widowed, separated, or never married, and 27.4% were married. Most participants perceived their current physical well-being as fair (16.4%), good or excellent (82.2%), or poor (1.4%). Variables of interest were relatively normally distributed, with none displaying skewness or kurtosis. 

The mean score for social support was 36.59, revealing participants reported moderate social support. The spiritual well-being mean score was 38.11, indicating moderate spiritual well-being. For depressive symptoms, the mean score was 9.31. Using CES-D-SF categories, approximately 46.6% of participants could be classiﬁed as having high levels of depressive symptoms.

In bivariate analysis, depressive symptoms were not correlated with demographic variables, such as age, education, income, and marital status, and, thus, were not included as covariates in subsequent analyses. However, family income was positively correlated to spiritual well-being (r = 0.29, p < 0.05) and perceived physical well-being (r = 0.24, p < 0.05), and education was negatively correlated with social support (r = -0.27, p < 0.05). Table 1 presents bivariate correlations between variables of interest. Importantly, depressive symptoms were negatively correlated with perceived physical well-being, spiritual well-being, and social support. 

*Multivariate Results*

Table 2 summarizes results from hierarchical regression analyses of protective factors against depressive symptoms. In the first model, perceived physical well-being was significant and explained 25% of the variance in the dependent variable (adjust R^2^ = 0.25, p < 0.001). Next, spiritual well-being was added, significantly related to depressive symptoms, and explained an additional 16% of variance (∆R^2^ = .16, p < .001). Finally, social support was added and explained an additional 7% of variance (∆R^2^ = 0.07, p < 0.01). In total, these three variables explained 47% of the variance in depressive symptoms (adjust R^2^ = 0.47). In the final model, perceived physical well-being (β = -0.35, p < 0.001), spiritual well-being (β = -0.27, p < 0.05), and social support (β = -0.31, p < 0.01) were negatively associated with depressive symptoms.

**Table 1:** Bivariate Correlations, Means, and Standard Deviations for Variables in the Models (N=73)

	1	2	3	4
1. Perceived physical well-being	--			
2. Spiritual well-being	0.32**	--		
3. Social support	0.22	0.53**	--	
4. Depression	-0.50**	-0.54**	-0.54**	--
Mean	2.52	38.11	35.92	9.32
SD	0.84	7.44	11.54	5.48

Notes. *p < 0.05. ** p < 0.01.

**Table 2:** Summary of Hierarchical Regression Analysis for Variables Predicting Depression (N = 73)

Variables	Model 1			Model 2			Model 3		
	B	SE B	β	B	SE B	β	B	SE B	β
Perceived physical well-being	-3.33	0.68	-0.51***	-2.44	0.63	-0.37***	-2.3	0.6	-0.35***
Spiritual well-being	.			-0.31	0.07	-0.43***	-0.2	0.08	-0.27*
Social support							-0.15	0.05	-0.31**
Adjusted R^2^			0.25			0.4			0.47
R^2^ Change			0.26			0.16			0.07
F Change			24.34***			19.35***			9.27**

Notes. *p < 0.05; **p < 0.01; ***p < 0.001

## Discussion

This exploratory study assessed how perceived physical well-being, spiritual well-being, and social support relate to depressive symptoms among AI female cancer survivors. Overall, approximately 47% of participants reported scores that exceed the cut off point for probable depression. This finding is similar to other studies (Gerbi et al., 2018; Hann et al., 2002) that found that depression was a common problem among cancer survivors in the general population. For example, Hann et al., (2002) reported that approximately 25% of cancer patients had clinical depression at outpatient clinics in Iowa, Wisconsin, Minnesota, and Georgia (Hann et al., 2002). However, in our study of AI women, rates of probable depression were 22% higher, highlighting the importance of identifying malleable factors that could be targeted to promote resilience within this population. 

The main results of the present study were that perceived physical well-being, spiritual well-being, and social support were inversely related to depressive symptoms, findings that are consistent with emerging research with the general population and the limited AI population studies (Bauer et al., 2005; Hann et al., 2002; Roh et al., 2015).

In terms of perceived physical health, our findings indicate that participants who reported lower health status were more likely to report probable depression compared with those with more favorable health status. The experience of surviving cancer is often replete with a series of severe stressors to various aspects of health (e.g., fear of death, body image, self-esteem, social roles, life plans) (Prieto et al., 2005). Despite living through a severe threat to one’s physical health (and mortality), there are likely lingering doubts about future threats, including recurrence. Thus, AI women who manage to retain a sense of physical vitality (e.g., renewed activity, acceptance of body changes, rebuilding of stamina) in the aftermath of cancer treatment may feel more efficacy and empowerment, which possibly buffer against depressive symptoms.

Results also indicated that spiritual well-being can protect against depressive symptoms among AI women cancer survivors. This finding is consistent with the few studies that have documented the positive effects of spirituality on depressive symptoms among AI cancer patients (McKinley et al., 2020; Roh et al., 2018). According to McKinley and colleagues (2020), AI cancer survivors coped in various traditional AI spiritual practices, such as smudging, ceremonies, and sweat lodges. Indeed, spiritual well-being has been found to be highly relevant for cancer treatment and care (Roh et al., 2018). According to Roh and associates’ (2018) qualitative study, majority of participants (76%, n = 32) noted prayer as an essential strategy for coping with cancer, management, and recovery. Many participants expressed how prayer and spirituality tied them to family, to faith communities, and to others, possibly leading to improved outlook and quality of life. 

Another finding was that greater social support was associated with lower depressive symptoms in this population. Social support may be especially protective for AI women cancer patients, considering the importance of connectedness and family and community support in AI sub-populations (Lee et al., 2018). Previous research has documented the centrality of family in the lives of AI populations, as well as the salience of prayers from fellow church members and companionship and advice from friends (Lee et al., 2018). Our study found that this support may shield cancer survivors from depressive symptoms long after medical cancer treatment and should be considered essential targets for comprehensive, effective, long-term treatment. 

Given the high prevalence of probable depression in this sample (47%), healthcare practitioners serving AI female cancer survivors should continue to assess for mental health problems long after treatment concludes. Specific training for health professionals to identify mental health problems among these populations may be needed. Additionally, multivariate results indicate promising pathways toward the amelioration of mental health disparities, such as depression in AI female cancer survivors, and have important implications for practice and future research. 

Health and mental health providers should understand the roles of physical and spiritual well-being and social support in this population. Social support and spirituality are protective factors that can be incorporated into culturally congruent practices with AIs, an approach consistent with patient-centered healthcare introduced nearly two decades ago (Institute of Medicine, 2001). A major impediment to culturally congruent practice with AI has been the lack of comprehensive and precise measures of spirituality in mental healthcare. Recently, several spiritual assessment tools have been validated with AIs and are readily available for use, such as spiritual histories, lifemaps, genograms, and eco-maps (Hodge and Limb, 2010a, 2010b). Furthermore, service providers must be mindful of the context of historical oppression and potential mistrust of formal services by AIs. Rather than formal services, many AI female cancer survivors prefer to rely on social networks, including extended families and friends, for instrumental and emotional support (Lee et al., 2018). Therefore, service providers may bolster informal networks and foster connections and social support to honor these preferences (Roh et al., 2015). 

Finally, with positive perceived physical health predicting lower depressive symptoms in this study, the connection between mental and physical health in the aftermath of cancer treatment is apparent. The physical health status of AI female cancer survivors should continue to be monitored during recovery and remission periods. Furthermore, an indigenous wellness model that incorporates mental, physical, and spiritual domains of health may serve to examine these connections holistically, in a culturally sensitive way.

Despite experiencing historical oppression and mental health disparities, AI female cancer survivors continue to demonstrate notable resilience. AI female cancer survivors have survived and thrived by relying on indigenous cultural resources, such as Native American spirituality and strong social support from family and community, and maintaining physical health in the aftermath of cancer treatment. Future qualitative research could advance our understanding of the ways in which this resilience is operationalized. Results could then be integrated into to culturally congruent interventions to assist AI cancer survivors who are experiencing depressive symptoms. 

The present study has several limitations. First, given its cross-sectional design and nonprobability sampling strategies, the present study focused on volunteer samples of AI female cancer survivors from two communities. As such, findings are only suggestive and require replication. Furthermore, selection bias might have affected findings in several ways. Participants might have been more willing to discuss the cancer experience and depressive symptoms than AI female cancer survivors who did not choose to participate. Future studies should also include a more representative sample and diverse tribal groups because cancer care may vary significantly by tribal affiliation, regional location, and rural/urban contexts. 

Additionally, the instrument that we used to measure spiritual well-being was originally designed to measure religiosity in Western religions, not AI tribes. Although the FACIT-Sp-12 Scale (Cellar et al., 1993) has been used extensively with diverse populations, it does not adequately assess the breadth and depth of AI cultural and spiritual traditions, such as the AI practice of dual spirituality, whereby traditional beliefs are fused with Western religious values (e.g., Christianity). Culturally grounded tools to assess spirituality or wellness might be useful in future research with this population. 

In spite of these limitations, the present study is one of the first studies to investigate important protective factors against depressive symptoms in AI female cancer survivors. Clinicians should consider assessing and promoting perceived physical well-being, spiritual well-being and social support as they work with cancer survivors with probable depression. According to Roh et al., (2018), AI female cancer survivors manage depressive feelings in a variety of ways, ranging from participation in faith traditions identifying creative and positive outlets, and consolidating family and social support. Based on our results, bolstering interpersonal (e.g., social support) and intrapersonal (e.g., spiritual health) activities appear to promising opportunities for improving cancer care and treatment. Also, physical health screening and promotion among AI women seem imperative. A community-based culturally appropriate health education program should be developed to increase AI women’s physical health. 

## Author Contribution Statement

The authors confirm contributions to the paper as follows: conception and design: SR, YSL; data collection: SR, YSL; analysis and interpretation of results: YPH, SR, YSL, SDE; draft manuscript preparation: SR, YSL, YPH, SDE.
